# Evolutionary Game Theoretic Analysis of Low Carbon Investment in Supply Chains under Governmental Subsidies

**DOI:** 10.3390/ijerph15112465

**Published:** 2018-11-05

**Authors:** Guang Zhu, Gaozhi Pan, Weiwei Zhang

**Affiliations:** 1 School of Management Science and Engineering, Nanjing University of Information Science and Technology, Nanjing 210044, China; 20161307043@nuist.edu.cn; 2 China Institute of Manufacturing Development, Nanjing University of Information Science and Technology, Nanjing 210044, China; zhangww@nuist.edu.cn

**Keywords:** evolutionary game, low carbon investment, supply chain, governmental subsidies, free riding

## Abstract

With the rapid development of global industry and economy, excessive carbon dioxide emission has emerged as a critical issue in both developed and developing countries. Using an evolutionary game framework in which game players can adjust their strategies constantly, this paper investigates how to optimize the strategy of low carbon investment for suppliers and manufacturers in supply chains, and discuss the impacts of various factors on evolutionarily stable strategies. Additionally, we examine an incentive mechanism based on governmental subsidies to eliminate free riding and motivate co-investment. Furthermore, a case study and numerical examples are provided for illustration and simulation purposes, leading to several countermeasures and suggestions. Our analytical results show that the strategic choice of low carbon investment is correlated with profit growth coefficients, investment costs and profits from free riding. Investment costs have more significant impacts than other factors on evolutionarily stable strategies, while profit growth coefficients are more important at initial stages in the evolutionary process. The incentive mechanism based on governmental subsidies is an effective solution to motivate co-investment, and governments should take some measures to improve the assess accuracy and supervisory efficiency of investment strategy.

## 1. Introduction

With the rapid development of the global industry and economy, the continuous population growth and excessive energy exploitation have caused serious environmental concerns, among which, global warming due to excessive carbon emissions has aroused attention worldwide [1,2]. In order to improve ecological conditions and promote the sustainable development of the economy, a low carbon development strategy, which includes a low carbon economy, low carbon industry, low carbon technology, and low carbon consumption, has been proposed in many countries [3].

Now, many governments and international organizations around the world have established various policies and protocols based on low carbon development. The European Commission proposed its low carbon economy roadmap, which suggested that the EU should cut its emissions to 80% below 1990 levels through domestic reductions alone by 2050 [4]. In the United Kingdom, the Climate Change Act 2008 outlined a framework for the transition to a low carbon economy, and required a cut in the UK’s carbon emissions by 2050, with an intermediate target of between 26% and 32% by 2020 [5]. Australia has implemented the Mandatory Renewable Energy Target (MRET) scheme, which requires 20% of Australia’s electricity supply to come from renewable energy sources by 2020 [6]. China has established the National Coordination Committee on Climate Change and taken a series of policies and measures to address climate change in the overall context of a national low carbon and sustainable development strategy. For example, the report “The improved response to climate change—China’s national independent contribution activities” stated that, by 2020, the concentration of carbon dioxide per unit of GDP will be 40–45% lower than in 2005 [7].

According to the above policies, we can conclude that governments’ regulation and guidance play important roles in low carbon development strategies. Meanwhile, active participation of the enterprises also has significant impacts on sustainable development and environmental conservation, because manufacturing is one of the main sources of carbon emissions [8]. Therefore, low carbon investment is envisioned to help enterprises stay updated and informed about environmental incidents. This allows them to develop various measures of low carbon development, such as the reduction of carbon emissions, the implementation of low carbon technologies, and the motivation of low carbon consumption [9,10]. However, enterprises still hesitate to invest in low carbon development for different reasons:

- The investment may not provide competitive advantages and extra profit in the market;
- An insufficient budget is viewed as the main challenge for low carbon investment [11];
- The investment process might create a channel that allows other entities to receive a free ride on low carbon expenditures, especially in manufacturing supply chains [12,13].

According to the above analysis, we know that financial factors significantly influence the strategic choice of low carbon investment, while the maximization of profit is considered the most common objective of business [14]. Hence, interest in studying how to optimize the strategy of low carbon investment with the consideration of profit and costs has aroused significantly.

Game theory provides a quantitative decision framework that can balance the profit and cost of low carbon investment [15]. Game theory assumes each player will choose the optimal strategic choice for profit maximization, which leads to the concept of equilibrium in a game [16,17]. In recent years, many game theoretic approaches have been implemented worldwide to address low carbon investment problems. However, existing research has several limitations, as follows:

(1) Current studies primarily consider the interactions between governments and enterprises and investigate the effects of low carbon subsidies and taxes [18,19,20]. However, research seldom focuses on the cooperation and interactions of low carbon behaviors among enterprises, which has been proved to be more important [4,21].
(2) Low carbon investment studies based on the classical game assume that game players are rational [22,23,24,25]. These game theoretic approaches ignore the dynamic process of behavior adaptations. It is difficult for players to achieve an optimal strategy in a single game process.
(3) Low carbon investment, which should be the only choice in practice, is indispensable for all enterprises. Economic factors that have impacts on strategic choices and the development of an incentive mechanism to motivate co-investment should be analyzed. Furthermore, there are several economic factors that influence the strategic choice of game players. Which factor has more significant impacts? Do the impacts of these factors stay consistent throughout the process of low carbon investment? Existing research has seldom considered these characteristics.

Considering the above limitations, the repeated game might provide a solution that allows for the study of immediate gains and long-term profits, which consists of a number of repetitions of some base game [26]. In a repeated game, a player should take into account the impact of his current action on the future actions of other players [27]. However, in low carbon investment, players are assumed to have bounded rationality due to having incomplete information and a complex decision-making process. Bounded rationality is the idea that when individuals make decisions, their rationality is limited by the tractability of the decision problem, the cognitive limitations of their minds, and the time available to make the decision [28]. Decision-makers, in this view, act via an evolutionary process and seek a satisfactory solution rather than an optimal one. Inspired by the behavior evolutionary process, this paper applies an evolutionary game theoretical approach to investigate the optimal strategy for low carbon investment. We take the manufacturing supply chains as the context and example for illustration and derive the evolutionarily stable strategies (ESSs) of suppliers and manufacturers. The study also proposes an incentive mechanism based on governmental subsidies to help motivate the local carbon investment. Finally, we use a numerical simulation to illustrate and validate the mathematical model and propose several countermeasures.

The rest of this paper is organized as follows: in Section 2, we review studies that are of relevance. Section 3 describes the methodology of this paper and proposes the research framework. Section 4 introduces the evolutionary game model and illustrates the ESSs under different constraints. Moreover, the impacts of different factors on ESSs are discussed, and an extended model with governmental subsidies is established. Section 5 verifies and analyzes the theoretical results obtained from numerical examples. Section 6 briefly summarizes our findings and provides some future research directions. All proofs are relegated to the appendices.

## 2. Literature Review

Much research effort has been spent discussing the impacts of different factors on low carbon strategies, and modelling different types of low carbon behavior. To better understand the motivation and roadmap of this paper, we divided the current studies into three categories: (1) the impacts of different factors on low carbon strategies; (2) classical game theoretic approaches to studying different types of low carbon behavior; and (3) evolutionary game theoretic approaches to studying low carbon behavior.

### 2.1. Impacts of Different Factors on Low Carbon Strategies

There has been substantial progress in the study of low carbon strategies that help to improve ecological conditions and promote sustainable development. Considering the inevitable trend of a low carbon economy, an integrated power generation expansion (PGE) planning model towards a low carbon economy is proposed, which properly integrates and formulates the impacts of various low carbon factors on PGE models [29]. In [30], a novel model to reduce carbon emission is proposed, which takes into account the link between an inventory policy (EOQ), total carbon emissions, and both price and environmental dependent demands. Considering the importance of supplier evaluation of carbon emissions, an integrated approach is presented by using fuzzy-AHP and fuzzy multi-objective linear programming. This research considers the impacts of various factors on selecting the appropriate supplier, including the cost, quality rejection percentage, late delivery percentage, greenhouse gas emissions, and demand [31]. Based on an analysis of the attitude-formation process, a study of the individual key consumption conditions from the cognitive process, to the affective process, and to low carbon behavioral choices was carried out. The results showed that the functional consumption value and the economical consumption value have significant influences on low carbon consumption cognition. The social consumption value was shown to significantly influence the preference for low carbon consumption preferences to others and low carbon consumption behavioral tendencies [32]. In [33], the path analysis method was used to explore the mechanisms of low carbon behavior effects on the direct energy use and related carbon emissions of households. The results showed that socioeconomic conditions combined with low carbon behaviors have direct and indirect impacts on household carbon emissions. Low carbon behavior in high-risk areas contributed most to reducing carbon emissions. The education level was the important factor in low-risk areas. Considering the important role of low carbon tourism in carbon emission reduction and environmental protection, a non-linear programming model was established on the basis of individual risk preferences, and a low carbon tourism destination selection case study was presented to illustrate how to use the decision-making model in practice [34]. In [35], the path analysis method was used to explore the mechanisms of low carbon behavior effects on the direct energy use and related carbon emissions of households. The results showed that socioeconomic conditions combined with low carbon behaviors have direct and indirect impacts on household carbon emissions. Low carbon behavior in high-risk areas contributed most to reducing carbon emissions. The education level was the important factor in low-risk areas. Considering the important role of low carbon tourism in carbon emission reduction and environmental protection, a non-linear programming model was established on the basis of individual risk preferences, and a low carbon tourism destination selection case study was presented to illustrate how to use the decision-making model in practice.

### 2.2. Classical Game Theoretic Approaches to Studying Low Carbon Behavior

As already stated, interest in studying how to optimize low carbon strategies with the consideration of different factors has aroused significantly. Game theoretic approaches provide a quantitative decision framework for modeling, analyzing, and predicting the behaviors of different participants. Some scholars have used game theory to explore low carbon problems and have obtained valuable research results. In the context of green supply chain management, a game theoretic approach was proposed to analyze the strategies selected by manufacturers. Through the application of the “tolerability of risk” concept, a basis for determining the extent of environmental risk and carbon emissions reduction was established [36]. In [37], the coordination between enterprises and suppliers was investigated with Bayesian game theory. Moreover, the impact of decentralized and centralized supply decisions on total carbon emissions was analyzed from a carbon footprint perspective. To reveal the underlying logic by analyzing the behaviors of the building owners and occupiers, the Nash Equilibrium of the game was used to analyze the probable decisions of the owners and occupiers under three scenarios: owner-occupied, single-occupied, and multi-occupied [38]. To ensure the sustainable energy consumption of firms and consequently improve the total social welfare, a game theoretical model was applied to analyze how a manufacturer’s operational decisions on sustainable energy consumption and low carbon production change with the variation in official cap-and-trade policies [39]. In [40], a supply chain game model was developed to analyze the impact of government subsidies on social welfare and on the profits of members of the supply chain. Considering the subsidy and tax as exogenous variables, a competitive game model for green and non-green products was developed to investigate the pricing policies, greening strategies, and governance carbon tax decisions in supply chains [41]. In the decentralized and centralized cases, a three-stage game model was proposed to investigate the influence of governmental carbon emission tax policies on manufacturers and suppliers in the supply chains [42]. Considering three dimensions—economic, social and environmental sustainability—a game model consisting of a supply chain and government was developed to discuss the effect of a government utility function on the supply chain members’ profits and on the green degree of the product [43]. Considering the competitive supply chain which consists of a manufacturer and a retailer, the pricing and carbon emission reduction decisions were discussed from the horizontal and vertical directions based on game theory [44]. In [45], a three stage game theoretic model was established to analyze the impact of government fiscal intervention on the competition within a green supply chain.

From the discussion above, we can see that most game theoretic research assumes there is a single scenario and investigates low carbon behavioral strategies based on Bayesian, Stackelberg, and differential game models. However, it is difficult to achieve an optimal strategy in a single game scenario where there is incomplete information and bounded rationality. Additionally, the individual game of low carbon behavior is a random and shared learning repetitive game process; thus, the adjustment process of individuals’ strategies can be simulated using a replicator dynamics mechanism. Hence, the evolutionary game model’s hypothesis of bounded rationality and a dynamic adjustment process appears to have more realistic significance [46].

### 2.3. Evolutionary Game Theoretic Approaches to Studying Low Carbon Behavior

In an ideal situation, the low carbon behaviors of governments, suppliers, and manufacturers occur under common knowledge of rationality. In reality, however, all participants are limited in rationality due to information asymmetry and market dynamics [47]. They have difficulty finding the optimal strategies at the outset, but improve their initial strategies through trial and error [48]. Evolutionary game theory does not rely on rationality assumptions but on the idea that evolutionary forces similar to, or such as, natural selection and mutation are the driving forces of change. It is better equipped to reflect the adjustment process of low carbon behavior. Considering the impacts of various combinations of carbon taxes and subsidies, an evolutionary game theoretic model was applied to discuss the behavioral strategies of the manufacturers and governments [49]. In the context of a complex network, an evolutionary game model of the low carbon strategies between the governments and enterprises is proposed. This research explored the effects of government incentives on enterprises regarding the diffusion of low carbon policies and how enterprises compete and transform in the Newman–Watts small-world network [50]. In [51], an evolutionary game model was proposed to investigate the possible responses of enterprises to incentive policies related to the implementation of a carbon reduction labeling scheme, such as a direct subsidy and preferential taxation rates. Considering four government subsidy strategies, an evolutionary game model was established to discuss the behavior of heterogeneous agents, including enterprises and consumers. In [52], an evolutionary game theoretic approach was proposed to analyze the impacts of a carbon quota, carbon trading price, government supervision cost, and enterprise emission reduction investment on the evolutionary stable strategies. To achieve green and low carbon development, an evolutionary game model of government and power producers based on carbon trading was constructed. The evolutionarily stable strategies were simulated by establishing a system dynamics (SD) model. Based on the simulation results, this research discussed the influence of government-controllable key factors on system stability [53]. Considering three different objective functions, an evolutionary game model of government and producers was established to analyze the influence of government policies on producer behavior and carbon emissions [46].

The literature review above demonstrates that most previous evolutionary game theoretic studies have considered the interactions between governments and enterprises, and investigated the impacts of governmental policies on the low carbon behaviors of enterprises. However, governmental regulation, tax, and subsidy are external factors. The low carbon investment of enterprises, especially the upstream suppliers and downstream manufacturers, is more important. On the one hand, the low carbon investment of suppliers ensures that the manufacturers can obtain low carbon raw materials and reduce costs. On the other hand, the low carbon investment of manufacturers influences the preference for, consumption of, and market requirement for low carbon products as well as the suppliers’ profits. Compared with the existing research, a significant difference in this paper is that it explores the decision-making processes of low carbon investment for suppliers and manufacturers from the perspective of internal cooperation and evolution.

Based on previous research, this paper establishes a two-echelon chain, consisting of suppliers and manufacturers, to study the optimal strategies of low carbon investment in supply chains within the framework of the evolutionary game theory. Meticulous theoretic and experimental analyses are made to discuss the conditions under which strategies are ESSs, and the impacts of different economic factors on the strategic decisions. This paper mainly contributes to the following aspects:

(1) We build an evolutional game model to formulate cooperative (or free riding) interactions and bounded rational confrontations between suppliers and manufacturers.
(2) We investigate the ESSs of low carbon investment, and the relationship between the economic factors and strategic choices.
(3) We propose an extended model based on governmental subsidies to avoid free riding and to motivate low carbon investment.
(4) We propose a case study and construct simulation experiments to demonstrate the usefulness of the proposed model, and we conduct a sensitivity analysis to investigate the impacts of different factors on evolutionary trends and the convergent speed.

## 3. Methodology

**Figure 1:**
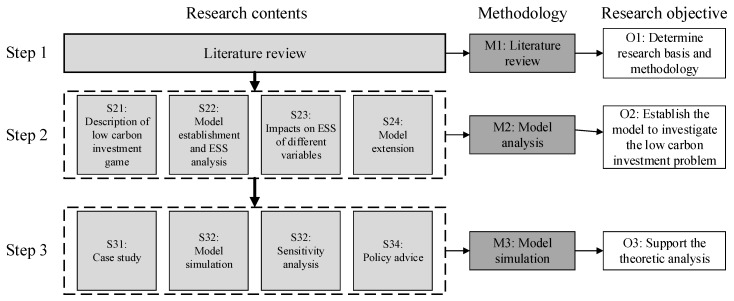
The research framework.

This paper adopted a hybrid research method including a literature review, model analysis, and numerical simulation. The research framework contained four mutually corresponding steps: Step 1, the literature review, provided the theoretical basis and methodological tools for research. Step 2, from S21 to S24, established an evolutionary game model to investigate the strategic choices involved in low carbon investment for suppliers and manufacturers, and to examine the different conditions under which strategies are ESSs. Additionally, the impacts of different variables on ESS were discussed. To motivate co-investment, an extended model under a contract with incentive mechanism was proposed. A case study was used to simulate the evolutionary path of low carbon behavior in Step 3, based upon which several policy advices were discussed. The research framework is shown in Figure 1.

## 4. Evolutionary Game Model of Low Carbon Investment

According to the analysis above, an effective implementation low carbon mechanism requires the enterprises’ active participation and investment, especially in supply chains. Therefore, the factors that influence the enterprises’ strategic choices should be deeply investigated. As a typical supply chain of carbon emission, the low carbon investment of the battery industry, which includes the use of environmental-friendly material, battery storage and recycling, and new energy battery technology, is quite important for ecological protection [54]. With these initial impressions, we went to Suntech, a leading solar photovoltaic manufacturer located in Wuxi, China [55], and interviewed the manager in charge of the financial budget, the manager in charge of low carbon technology, and several engineers and salespeople. In addition, we also went to BYD (Bi Ya Di), a highly regarded supplier of batteries in Shenzhen, China [56], and interviewed the manager in charge of the financial budget and market, several engineers, and the relevant people. Through this investigation, we identified the key parameters for the evolutionary model.

### 4.1. Model Establishment

Based on the relationships involved in low carbon investment in supply chains, we first defined two types of evolutionary game players: suppliers (denoted by *S*) and manufacturers (denoted by *M*) Both of them have two strategic choices: “invest” and “not invest”. The low carbon investment of suppliers includes energy saving and emission reduction, renewable energy, carbon capture and storage, etc. The investment of manufacturers includes the purchase of low carbon materials, business process re-engineering, low carbon technology research, etc. [57]. As a result, there were four possible combinations of strategies: (not invest, not invest), (not invest, invest), (invest, not invest), and (invest, invest).

An evolutionary game models the strategic interactions over time in terms of one or more populations of players. However, the population of suppliers and manufacturers is not completely homogeneous in reality, because suppliers mighty not satisfy the requirements for the upgraded material of green technology. To facilitate the model’s establishment and solution, we assumed that each player has its game partner. In other words, any supplier can be matched with any manufacturer. Based on the analysis above, the investment strategies of suppliers and manufacturers during the adjustment and dynamic process have the salient features of an evolutionary game.

#### 4.1.1. Payoff Matrix

If none of the game players choose “invest”, the original profits of suppliers from selling raw material can be define as $B_{S}$. The original profits of manufacturers are defined as $B_{M}$.

In the supply chains of low carbon investment, suppliers invest $C_{S}$ for energy saving, renewable energy and offer low carbon raw material to manufacturers. On the other hand, manufacturers invest $C_{M}$ for low carbon process re-engineering and low carbon technology research. In order to cover investment costs, the prices of low carbon raw material and products are higher. In this paper, we assume that consumers who have sustainable consciousness are willing to pay more for low carbon products.

If only suppliers choose “invest”, carbon emissions can be reduced upstream of supply chains. Suppliers will provide low carbon material to manufacturers, and consumers are willing to pay more for low carbon products. Therefore, the profits from low carbon investment can be defined as $(1+\alpha_{0})B_{S}-C_{S}$, where $\alpha_{0}(\alpha_{0}>0)$ is the profit growth coefficient. Similarly, if only manufacturers choose “invest”, carbon emissions can be reduced downstream of supply chains. Consumers are also willing to pay more for low carbon products. The profits from low carbon investment can be defined as $(1+\beta_{0})B_{M}-C_{M}$, where $\beta_{0}(\beta_{0}>0)$ is the profit growth coefficient. If both participants choose “invest”, the low carbon development strategy can be implemented at a higher level. Consumers are willing to pay more than in the last scenario. The profits of suppliers and manufacturers can be defined as $(1+\alpha_{1})B_{S}-C_{S}$ and $(1+\beta_{1})B_{M}-C_{M}$, respectively, where $\alpha_{1}>\alpha_{0}>0$ and $\beta_{1}>\beta_{0}>0$.

In the evolutionary game model, the strategic choices of game players have significant impacts on the other side. Therefore, the behavior features of free riding should be considered. If only suppliers invest in low carbon development upstream of supply chains, manufacturers can get the low carbon material and claim that the product is green and environmentally friendly. They can sell their products at a higher price and obtain extra profits from free riding, without any investment costs. Additionally, their free riding behavior cannot be recognized precisely due to limited budgets and technological support.

On the other hand, if only manufacturers invest in low carbon technology and green engineering downstream of supply chains, the products can also be improved and sold at a higher price. Our interview of several consumers found that they believe that low carbon raw material plays a significant role in the final products. Therefore, we can conclude that suppliers obtain recessive profits by free riding the manufacturers’ investment, such as market reputation and advertisement effects.

The profits from the free riding of suppliers and manufacturers can be defined as $\xi_{S}$ and $\xi_{M}$, respectively. Most of the key notations that occur in this paper are listed in Table 1 for easy reference.

Additionally, we assume game players have the same level of bargaining power. Furthermore, the variation of model parameters is relatively small during the evolutionary process and has no significant impact on the evolutionary trend.

Considering the cooperative relationship between suppliers and manufacturers, the payoff matrix is shown in Table 2.

#### 4.1.2. Equilibrium Analysis

In the initial stage of the evolutionary game, x(0≤x≤1) is defined as the population of suppliers making the strategic choice of “invest”. In contrast, 1−x represents the population making the strategic choice of “not invest”. Similarly, y(0≤y≤1) represents the population of manufacturers making the strategic choice of “invest”, and 1−y represents the population making the strategic choice of “not invest”.

Based on the payoff matrix, it is assumed that $\mu_{1,1}$ represents the expected payoff of suppliers that make the strategic choice of “invest”, $\mu_{1,2}$ represents the expected payoff of suppliers that make the strategic choice “not invest”, and $\mu_{1}$ represents the average expected payoff. Therefore:

(1) $$\mu_{1,1}=y[(1+\alpha_{1})B_{S}-C_{S}]+(1-y)[(1+\alpha_{0})B_{S}-C_{S}]$$

(2) $$\mu_{1,2}=y\xi_{S}+(1-y)B_{S}$$

Thus, the average expected payoff can be written as follows:

(3) $$\mu_{1}=x\mu_{1,1}+(1-x)\mu_{1,2}$$

Similarly, it is assumed that $\mu_{2,1}$ represents the expected payoff of manufacturers that make the strategic choice of “invest”; $\mu_{2,2}$ represents the expected payoff of manufacturers that make the strategic choice of “not invest”; and $\mu_{2}$ represents the average expected payoff. Therefore:

(4) $$\mu_{2,1}=x[(1+\beta_{1})B_{M}-C_{M}]+(1-x)[(1+\beta_{0})B_{M}-C_{M}]$$

(5) $$\mu_{2,2}=x\xi_{M}+(1-x)B_{M}$$

(6) $$\mu_{2}=y\mu_{2,1}+(1-y)\mu_{2,2}$$

According to the Malthusian dynamic equation [58], the replication dynamic equation of suppliers is:

(7) $$G(x)=\frac{dx}{dt}=x(\mu_{1,1}-\mu_{1})=x(1-x)\{\alpha_{0}B_{S}-C_{S}-[\xi_{S}-(\alpha_{1}-\alpha_{0}+1)B_{S}]y\}$$

The replication dynamic equation of manufacturers is:

(8) $$G(y)=\frac{dy}{dt}=y(\mu_{2,1}-\mu_{2})=y(1-y)\{\beta_{0}B_{M}-C_{M}-[\xi_{M}-(\beta_{1}-\beta_{0}+1)B_{M}]x\}$$

When the dynamic equations equal 0, an equilibrium point has been reached, and the equations will no longer evolve. This results in five equilibrium points: (0, 0), (0, 1), (1, 0), (1, 1), and (*A*, *B*). The term (*A*, *B*) is a mixed equilibrium point where $A=\frac{\beta_{0}B_{M}-C_{M}}{\xi_{M}-(\beta_{1}-\beta_{0}+1)B_{M}}$, $B=\frac{\alpha_{0}B_{S}-C_{S}}{\xi_{S}-(\alpha_{1}-\alpha_{0}+1)B_{S}}$. The stability of equilibrium points can be analyzed using the Jacobian matrix as follows:

(9) $$J=\left[\begin{array}{l} \frac{\partial G(x)}{\partial x}\frac{\partial G(x)}{\partial y}\\ \frac{\partial G(y)}{\partial x}\frac{\partial G(y)}{\partial y} \end{array}\right]=\left[\begin{array}{l} a_{11}a_{12}\\ a_{21}a_{22} \end{array}\right]$$

Therefore, we can compute the values of equilibrium points that are shown in Table 3.

When the equilibrium point satisfies trJ<0 and detJ>0, this equilibrium point is an ESS. We can find that (A,B) is not satisfied under these conditions because $a_{11}+a_{22}=0$. Other equilibrium points will be ESSs, whereas the values of $\alpha_{0}$, $\alpha_{1}$, $\beta_{0}$ and $\beta_{1}$ are satisfied under different conditions. The propositions are elaborated as follows:

**Proposition** **1.** *When the profit growth coefficients of low carbon investment are small, which are given as*$0<\alpha_{0}<\frac{C_{S}}{B_{S}}$*,*$\alpha_{0}<\alpha_{1}<\frac{\xi_{S}+C_{S}-B_{S}}{B_{S}}$*and*$0<\beta_{0}<\frac{C_{M}}{B_{M}}$*,*$\beta_{0}<\beta_{1}<\frac{\xi_{M}+C_{M}-B_{M}}{B_{M}}$*, (0, 0) is an evolutionarily stable point, while suppliers and manufacturers will not invest because of the low profit.*

**Proof.**  See Appendix A. □

In this case, the increased profits from low carbon investment $\left(\alpha_{0}B_{S},\beta_{0}B_{M}\right)$ are so small that they cannot even cover the investment costs $\left(C_{S},C_{M}\right)$. From a business perspective, consumers’ expenditure on low carbon products will remain fixed within a certain period. Therefore, the profit growth coefficients are decided by the investment costs. If the investment costs are high, both suppliers and manufacturers are willing to choose “not invest”, especially when there are no governmental subsidies.

Proposition 1 also presents the business implications from the perspective of an evolutionary analysis. We assume there are several suppliers and manufacturers in supply chains. Supplier $s_{i}$ may choose “invest” at first because of information asymmetry and bounded rationality. Then, $s_{i}$ finds $s_{j}$ (another supplier) which chooses “not invest” and gets higher profits. Therefore, $s_{i}$ adjusts and improves its choices by imitating the strategy of $s_{j}$ for profit maximization. We can conclude that the strategy of $s_{j}$ will have an impact on the strategic decisions of $s_{i}$. Moreover, the investment strategies of manufacturers also impact the strategic decisions of suppliers. Their interactions with each other will result in the evolution of strategic choices.

**Figure 2:**
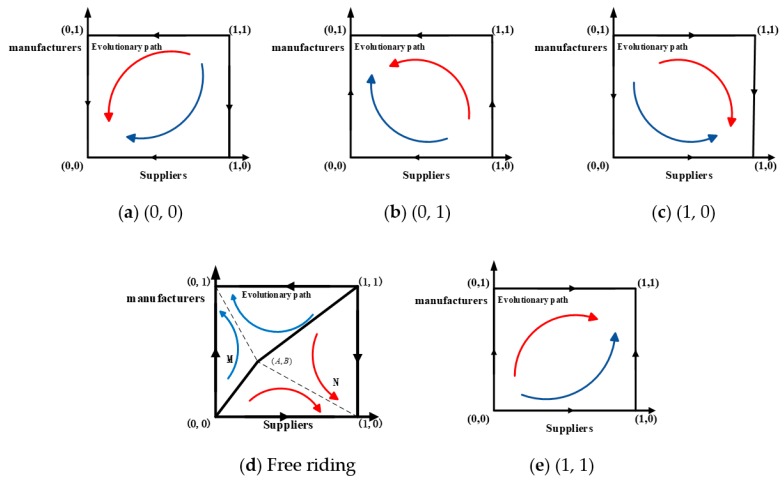
Evolution of the dynamic model.

Panel (a) in Figure 2 displays the evolution of the dynamic model when the profit growth coefficients are small. We can see that the evolutionary model will eventually converge at (0, 0) no matter which strategies are initially used by game players. Therefore, (0, 0) is the evolutionarily stable point; (0, 1) and (1, 0) are saddle points; and (1, 1) is the unstable point. The ESS profile is (not invest, not invest).

**Proposition** **2.** *As the profit growth coefficients of manufacturers increase, which are given as* $0<\alpha_{0}<\frac{C_{S}}{B_{S}}$ *,* $\alpha_{0}<\alpha_{1}<\frac{\xi_{S}+C_{S}-B_{S}}{B_{S}}$ *and* $\frac{C_{M}}{B_{M}}<\beta_{0}<\beta_{1}<\frac{\xi_{M}+C_{M}-B_{M}}{B_{M}}$ *, (0, 1) is an evolutionarily stable point, and manufacturers will prefer to invest to get higher profits, while supplier still choose “not invest” because of the low profits.*

**Proof.**  See Appendix B. □

In this case, the increased profits of the manufacturers from the low carbon investment $\left(\beta_{0}B_{M}\right)$ are higher than the investment costs. From the business perspective, the increased profits can be brought about by the reduced costs and by selling the low carbon products at a higher price. As stated already, the profits from free riding are only possible when at least one side of game players chooses “invest”. Therefore, manufacturers are willing to choose “invest” to give themselves a better reputation and profit maximization. From the perspective of evolutionary analysis, it is assumed that manufacturer $m_{i}$ may choose “not invest” at first because of the investment costs. Then, $m_{i}$ finds that $m_{j}$ chose “invest” and achieved higher profits. Therefore, $m_{i}$ will improve its choice by imitating the strategy of $m_{j}$. Moreover, the investment strategies of suppliers have no significant impacts on the strategic decisions of manufacturers, because manufacturers cannot free ride on the other side of game players.

Panel (b) in Figure 2 depicts the dynamic evolution model. As shown, the model will eventually converge at (0, 1) no matter which strategies are initially used by game players. Therefore, (0, 1) is the evolutionarily stable point; (0, 0) and (1, 0) are saddle points; and (1, 1) is the unstable point. The ESS profile is (do not invest, invest).

**Proposition** **3.** *As the profit growth coefficients of the suppliers increase, which are given as* $\frac{C_{S}}{B_{S}}<\alpha_{0}<\alpha_{1}<\frac{\xi_{S}+C_{S}-B_{S}}{B_{S}}$ *and* $0<\beta_{0}<\frac{C_{M}}{B_{M}}$ *,* $\beta_{0}<\beta_{1}<\frac{\xi_{M}+C_{M}-B_{M}}{B_{M}}$ *, (0, 1) is an evolutionarily stable point, and suppliers will prefer to choose “invest” to get higher profits, while manufacturers choose “not invest”.*

**Proof.**  See Appendix C. □

In this case, the increased profits of suppliers from low carbon investment $\left(\alpha_{0}B_{S}\right)$ are higher than the investment costs. From the business perspective, the increased profits can be brought about by the use of low carbon material. Therefore, suppliers are willing to choose “invest” to attract more consumers. From the perspective of evolutionary analysis, supplier $s_{i}$ may choose “not invest” at first because of bounded rationality. Then, $s_{i}$ finds that $s_{j}$ chose “invest” and achieved higher profits. Therefore, $s_{i}$ adjusts its strategic choice by imitating the strategy of $s_{j}$. Similarly, the investment strategies of manufacturers have no significant impacts on the strategic decision of suppliers, because suppliers cannot free ride on the other side of game players.

Panel (c) in Figure 2 illustrates the evolution of the dynamic model. The figure shows it will eventually converge at (1, 0) no matter what strategies are initially used by game players. Therefore, (1, 0) is the evolutionarily stable point; (0, 0) and (0, 1) are saddle points; and (1, 1) is the unstable point. The ESS profile is (invest, not invest).

**Proposition** **4.** *If both the profit growth coefficients of suppliers and manufacturers increase to a certain level, which are given as* $\frac{C_{S}}{B_{S}}<\alpha_{0}<\alpha_{1}<\frac{\xi_{S}+C_{S}-B_{S}}{B_{S}}$ *and* $\frac{C_{M}}{B_{M}}<\beta_{0}<\beta_{1}<\frac{\xi_{M}+C_{M}-B_{M}}{B_{M}}$ *, (0, 1) and (1, 0) are evolutionarily stable points. There are two ESSs, while suppliers and manufacturers are not sure whether they will choose “invest” or not. Game players will always adjust and improve their strategic choice during the evolution process because they want to free ride off others.*

**Proof.**  See Appendix D. □

In this case, both the increased profits of suppliers and manufacturers from low carbon investment $\left(\alpha_{0}B_{S},\beta_{0}B_{M}\right)$ are higher than the investment costs, but lower than the profits from free riding $\left(\xi_{S},\xi_{M}\right)$. From the business perspective, the increased profits can be brought about by reduced costs as well as technological development and market expansion, which was mentioned above. Moreover, free riding can bring higher profits due to its better reputation, greater number of consumers and low cost. From the perspective of evolutionary analysis, Supplier $s_{i}$ and manufacturer $m_{i}$ may choose “invest” at first because of the higher profits from low carbon investment. Then, $s_{i}$ finds that it can get higher profits if it can free ride off $m_{i}$. For example, if $m_{i}$ chooses “invest”, there will be more consumers to use low carbon products. Therefore, $s_{i}$ can get extra profits from a larger market without any investment costs. However, it is not the end of evolution process. $m_{i}$ will also choose “not invest” and want to free ride on $s_{i}$. Thus, $s_{i}$ and $m_{i}$ will always adjust their strategy by imitation for profit maximization.

Panel (d) in Figure 2 depicts the evolution of the dynamic model. As shown, the model will eventually converge at (0, 1) or (1, 0). Therefore, (1, 0) and (0, 1) are the evolutionary stable points; (A,B) is the saddle point; (1, 1); and (0, 0) are the unstable points. The ESS profiles are (not invest, invest) and (invest, not invest).

**Proposition** **5.** *As the profit growth coefficients of increase continually, which are given as* $\frac{\xi_{S}+C_{S}-B_{S}}{B_{S}}<\alpha_{0}<\alpha_{1}$ *and* $\frac{\xi_{M}+C_{M}-B_{M}}{B_{M}}<\beta_{0}<\beta_{1}$ *, (1, 1) is an ESS, both suppliers and manufacturers will choose “invest”.*

**Proof.**  See Appendix E. □

In this case, both the increased profits of suppliers and manufacturers from low carbon investment $\left(\alpha_{0}B_{S},\beta_{0}B_{M}\right)$ are not only higher than the investment costs $\left(C_{S},C_{M}\right)$, but also higher than the profits from free riding $\left(\xi_{S},\xi_{M}\right)$. Therefore, suppliers and manufacturers are willing to invest and can get appropriate profits, and (invest, invest) is the optimal strategy.

From the perspective of evolutionary analysis, even if $s_{i}$ or $m_{i}$ chooses “not invest” at first, they will find that “invest” can bring higher profits sooner or later. Therefore, both players will adjust their strategic choice by imitating others’.

Panel (e) in Figure 2 shows the evolution of the dynamic model. As shown, it will eventually converge at (1, 1) regardless of strategies initially taken by OSN service providers and online platforms. Therefore, (1, 1) is the evolutionarily stable point; (0, 1) and (1, 0) are saddle points; and (0, 0) is the unstable point. The ESS profile is (invest, invest).

#### 4.1.3. Impact of different variables on ESS

According to Proposition 4, the ESS can be either (invest, not invest) or (not invest, invest) when free riding is present. The strategic choice depends on the area sizes of regions *M* and *N* which can be written, as $\left(S_{M},S_{N}\right)$, respectively. The probability of choosing (invest, not invest) is greater if $S_{M}>S_{N}$, while the probability of choosing (not invest, invest) is higher if $S_{M}<S_{N}$. $S_{M}$. This can be defined as follows:

(10) $$S_{M}=\frac{1}{2}[\frac{\beta_{0}B_{M}-C_{M}}{\xi_{M}-(\beta_{1}-\beta_{0}+1)B_{M}}+\frac{\xi_{S}-(1+\alpha_{1})B_{S}+C_{S}}{\xi_{S}-(\alpha_{1}-\alpha_{0}+1)B_{S}}]$$

According to Equation (10), there are 10 variables that influence the ESS, and further conclusions can be drawn, as shown in Table 4.

**Conclusion** **1.** The smaller the profit growth coefficients of suppliers $\left(\alpha_{0},\alpha_{1}\right)$ are, the higher the probability of them converging to the equilibrium point (0, 1) is. In this case, suppliers and manufacturers will choose (not invest, invest). The larger the profit growth coefficients of suppliers are, the higher the probability of them converging to equilibrium point (1, 0) is. Suppliers and manufacturers will choose (invest, not invest).

**Proof.**  See Appendix F. □

**Conclusion** **2.** The smaller the profit growth coefficients of manufacturers $\left(\beta_{0},\beta_{1}\right)$ are, the higher the probability of them converging to equilibrium point (1, 0) is. In this case, suppliers and manufacturers will choose (invest, not invest). The larger the profit growth coefficients of manufacturers are, the higher the probability of them converging to equilibrium point (0, 1) is. Suppliers and manufacturers will choose (not invest, invest).

**Proof.**  See Appendix G. □

**Conclusion** **3.** The smaller the original profits of suppliers $\left(B_{S}\right)$ are and the larger the original profits of manufacturers $\left(B_{M}\right)$ are, the higher the probability of them converging to equilibrium point (0, 1) is. In this case, suppliers and manufacturers will choose (not invest, invest). The larger $B_{S}$ is and smaller $B_{M}$ is, the higher the probability of them converging to (1, 0) is, and they will choose (invest, not invest).

**Proof.**  See Appendix H. □

**Conclusion** **4.** The smaller the investment costs of manufacturers $\left(C_{M}\right)$ are and the larger the investment costs of suppliers $\left(C_{S}\right)$ are, the higher the probability of converging them to equilibrium point (0, 1) is. In this case, suppliers and manufacturers will choose (not invest, invest). The larger $C_{M}$ is and the smaller $C_{S}$ is, the higher the probability of them converging to (1, 0) is, and they will choose (invest, not invest).

**Proof.**  See Appendix I. □

**Conclusion** **5.** The smaller the profits of manufacturers from free riding $\left(\xi_{M}\right)$ are and the larger the profits of suppliers from free riding $\left(\xi_{S}\right)$ are, the higher the probability of them converging to equilibrium point (0, 1) is. In this case, suppliers and manufacturers will choose (not invest, invest). The larger $\xi_{M}$ is and the smaller $\xi_{S}$ is, the higher the probability of them converging to (1, 0) is, and they will choose (invest, not invest).

**Proof.**  See Appendix J. □

In summary, during the evolutionary process of low carbon investment for suppliers and manufacturers, the investment desire is affected by the original profits, investment costs, profit growth coefficients, and profits from free riding. Moreover, the evolutionary path of free riding and the final result of both investors’ decision-making processes are also affected by the initial strategic choices.

### 4.2. Model Extension

According to the equilibrium analysis above, if the profit growth coefficients satisfy $\frac{C_{S}}{B_{S}}<\alpha_{0}<\alpha_{1}<\frac{\xi_{S}+C_{S}-B_{S}}{B_{S}}$ and $\frac{C_{M}}{B_{M}}<\beta_{0}<\beta_{1}<\frac{\xi_{M}+C_{M}-B_{M}}{B_{M}}$, the profits from low carbon investment will be higher than the investment costs, but lower than the profits from free riding. Therefore, both suppliers and manufacturers want to free ride on the other side and prefer to choose “not invest”. This is not our expected ESS, as it could result in low efficiency for ecology conservation and sustainable development.

To eliminate free riding, an incentive mechanism based on governmental subsidies should be developed. In this paper, the incentive is expressed as the subsidy to the participant who invests in the low carbon strategy, which equals the fine for the player who does not invest. Moreover, the fine and subsidy are applied at the same time to reduce the regulation costs of the government.

To facilitate the model analysis, both the subsidy and fine variable are defined as K. The payoff matrix is shown in Table 5.

Similarly, we also can get five equilibrium points: (0, 0), (0, 1), (1, 0), (1, 1), (*A*′, *B*′). The term (*A*′, *B*′) is a mixed equilibrium point where $A^{\prime }=\frac{\beta_{0}B_{M}-C_{M}+K}{\xi_{M}-(\beta_{1}-\beta_{0}+1)B_{M}}$, $B^{\prime }=\frac{\alpha_{0}B_{S}-C_{S}+K}{\xi_{S}-(\alpha_{1}-\alpha_{0}+1)B_{S}}$.

The incentive mechanism based on governmental subsidies is developed to motivate suppliers and manufacturers to choose “invest” and eliminate free riding. The equilibrium point (1, 1) should be the unique stable point, and (invest, invest) is the optimal ESS from the perspective of sustainable development and environmental conservation. Therefore, we can conclude that K should satisfy the following condition:

(11) $$K>\max\{\xi_{S}-[(\alpha_{1}+1)B_{S}-C_{S}],\xi_{M}-[(\beta_{1}+1)B_{M}-C_{M}]\}$$

If *K* satisfies the condition above, the ESS (invest, invest) can be our expected result. It should be noted that the result would not dramatically change if either a fine or a subsidy is applied at a given time. For example, if a government just gives the subsidy, but does not charge for the fine, we can conclude that *K* should still satisfy the condition in Equation (11). The only difference is that the government needs to pay the regulation costs.

## 5. Case Study and Simulation Analysis

A case of two photovoltaic enterprises was used to simulate the evolutionary behaviors of suppliers and manufacturers under different conditions, which could theoretically be applied in many other manufacturing industries around the world which produce products that have no distinctly low carbon characteristics.

### 5.1. Case Study

Suntech is a leading solar photovoltaic manufacturer, which was established in 2001 and is located in Wuxi, China. BYD is a highly regarded supplier of batteries and new energy devices, which was established in 1995 and is located in Shenzhen, China. BYD provides solar batteries and photovoltaic power boards to Suntech, and Suntech sells photovoltaic application products to consumers. It is well-known that batteries contain a number of heavy metals and toxic chemicals. If we dispose batteries by the same process as regular trash, serious concerns are raised over soil contamination and water pollution. Therefore, low carbon technology investment for the production and recycling of batteries is quite important for environmental conservation.

The parameters of the evolutionary game model were estimated from an interview of these two firms and a survey of the market. To facilitate the model simulation, the parameter values were set by formulation and normalization, as follows: if neither Suntech nor BYD chooses to invest in low carbon development, the current price of solar battery can be defined as 60 CNY/pce, and the price of the photovoltaic product is 120 CNY/pce. Moreover, through our investigation and interview, we set the low carbon investment costs of BYD to 30 CNY, and the investment costs of Suntech to 48 CNY. Then, we assumed that the profits from free riding were $\xi_{S}=100$ CNY and $\xi_{M}=160$ CNY, respectively. The initial parameters for the simulation analysis are offered in Table 6.

Thus, we can calculate the following:

(12) $$\frac{C_{S}}{B_{S}}=0.5,\;\frac{C_{M}}{B_{M}}=0.4,\;\frac{\xi_{S}+C_{S}-B_{S}}{B_{S}}=1.17,\;\frac{\xi_{M}+C_{M}-B_{M}}{B_{M}}=0.73$$

Based on the critical points above, the government can then perform a numerical simulation to estimate the subsidies or fines to help reach the ESS of (invest, invest). Table 7 shows some examples of $\alpha_{0}$, $\alpha_{1}$, $\beta_{0}$, $\beta_{1}$ and their corresponding ESSs. Please note that the values of profit growth coefficients used in this table are just for illustration.

### 5.2. Simulation of Evolutionary Game Model

Our game equilibriums provide a detailed description of the game model and its properties. In this section, we describe the numerical results from our game analysis and use MATLAB R2010a (Mathworks, Natick, MA, USA) to simulate and support the game-theoretic analysis. We assigned fixed values to several variables; other variables increased or decreased relative to the assigned variables.

**Figure 3:**
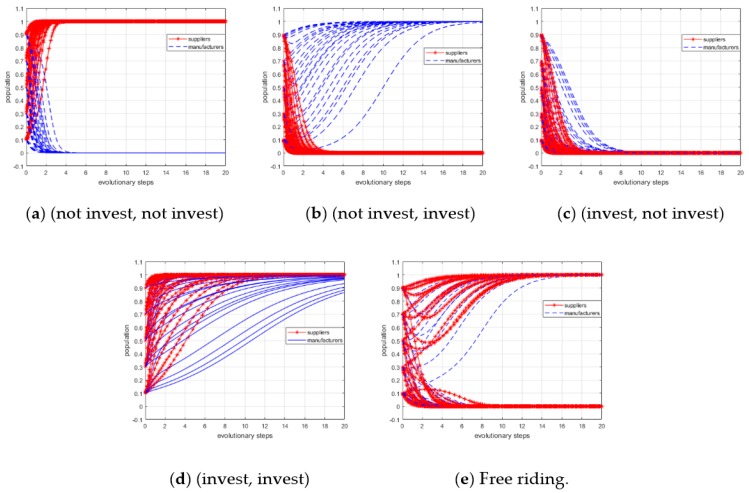
Simulation results of the basic model.

At first, we set $\alpha_{0}=0.25$, $\alpha_{1}=0.45$, $\beta_{0}=0.25$, and $\beta_{1}=0.45$, which satisfied the conditions $0<\alpha_{0}<\frac{C_{S}}{B_{S}}$, $\alpha_{0}<\alpha_{1}<\frac{\xi_{S}+C_{S}-B_{S}}{B_{S}}$, and $0<\beta_{0}<\frac{C_{M}}{B_{M}}$, $\beta_{0}<\beta_{1}<\frac{\xi_{M}+C_{M}-B_{M}}{B_{M}}$. The initial population (x, y) of suppliers (BYD) and manufacturers (Suntech) who chose “invest” was set from 10% to 90%. The simulation result is shown in panel (a) of Figure 3. In this scenario, the profit growth coefficients were relatively small; that is, the low carbon investment will not bring the expected profits to suppliers and manufacturers. Therefore, the population (x, y) will converge to zero, and the ESS is (not invest, not invest). This simulation result is consistent with Proposition 1.

When the profit growth coefficients are set to different values, as shown in Table 7, the simulation results are depicted in panels (b), (c), and (d) of Figure 3. The ESSs are (not invest, invest), (invest, not invest), and (invest, invest), respectively. Moreover, according to the analysis of Proposition 4, we know that there is no unique ESS if the profit growth coefficients satisfy the conditions $\frac{C_{S}}{B_{S}}<\alpha_{0}<\alpha_{1}<\frac{\xi_{S}+C_{S}-B_{S}}{B_{S}}$ and $\frac{C_{M}}{B_{M}}<\beta_{0}<\beta_{1}<\frac{\xi_{M}+C_{M}-B_{M}}{B_{M}}$. In this scenario, some of the game players will always want to obtain extra profits by free riding off other players. To verify this result, we set $\alpha_{0}$ = 0.6, $\alpha_{1}$ = 0.8, $\beta_{1}$ = 0.5, and $\beta_{1}$ = 0.7. The results of simulation are shown in panel (e) of Figure 3. The population (x, y) will not converge to a fixed value, instead it settles at either (0, 1) or (1, 0) depending on the initial state of the system and the values of the related variables.

**Figure 4:**
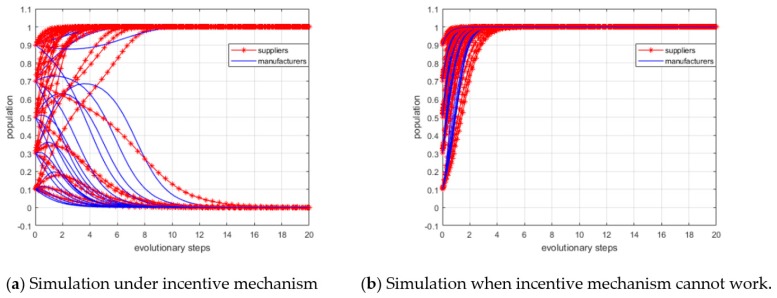
Simulation results of extended model.

According to the previous analysis, we know that if the profit growth coefficients are small and the other variables remain fixed, both suppliers and manufacturers will choose “not invest”, or free riding might be present. To motivate co-investment for better low carbon development, we developed an incentive mechanism based on governmental subsidies. Based on the analysis in Section 4, the term K should be satisfied by Equation (11). Thus, we obtained K>max{2.2,1.5}. We defined K=3.0, and the initial population was set from 10% to 90%. The simulation results are shown in panel (a) of Figure 4. With the inclusion of governmental subsidies, both suppliers and manufacturers are willing to choose “invest”, and none of them can earn extra profits through free riding. Therefore, the optimal ESS is obtained—(invest, invest).

In this paper, we assume that the investment decision of suppliers and manufacturers can be assessed by the governments, and the subsidy amount is enough. However, not all behaviors of low carbon investment or free riding can be recognized and evaluated precisely due to limited budgets and technological support. To investigate this scenario, we defined K=2.0 which cannot satisfy the condition given by Equation (11). The simulation result is shown in panel (b) of Figure 4. The strategic choices of manufacturers converged to “invest”. However, the strategic choices of suppliers cannot converge to the unique ESS. Thus, whether they invest in low carbon development or not is dependent on the initial state of the evolutionary system.

According to our interview and market survey of two photovoltaic enterprises, we can estimate that the range of profit growth coefficients in our case is $0.2<\alpha_{0}<\alpha_{1}<0.4$ and $0.18<\beta_{0}<\beta_{1}<0.33$. This indicates that neither the suppliers nor the manufacturers will choose “invest” due to the low profits. In this scenario, determining how to increase the minimum profit growth coefficients and governmental intervention are more important to the low carbon strategy. Based on the analysis above, we propose several policy advice items:

*Policy advice 1*. Both the suppliers and the manufacturers should increase their minimum profit growth coefficients. The policy-makers can create this condition by implementing the following measures:

(1) Enhancing the consciousness of low carbon development

Based on the model analysis and numerical simulation, the profit growth coefficients are the fundamental driving forces of low carbon investment. Therefore, proper environment conservation education programs should be developed and strengthened. This would broaden the consciousness about low carbon issues. Additionally, public lectures on sustainable development should be held, so domain experts can systematically teach an appropriate attitude towards low carbon economy and life. Through these measures, more consumers will be attracted and willing to pay more for low carbon products.

(2) Offering differentiated types of products

Suppliers and manufacturers should consider various factors, such as the economic conditions of consumers, living locations, and differences in environmental consciousness. All of these factors have significant impacts on consumers’ choice to pay more for low carbon products or not. To address this problem, suppliers and manufacturers can provide differentiated types of raw materials and products, for instance, the basic products can be offered at a low price to consumers, while the value-added products that offer improved levels of environment conservation and sustainable development are be provided at a higher price. Through these two-type mechanisms, suppliers and manufacturers can appropriately balance the profits and costs of low carbon investment.

(3) Awarding innovation in low carbon technology

It is obvious that the ESS (invest, invest) is the optimal strategic choice in practice. Therefore, governments should give priority to financially supporting or encouraging low carbon investment through tax incentives or financial subsidies. Moreover, any technological innovation related to low carbon development that can increase profits and reduce costs should be encouraged and motivated through national science and technology plans or industrial development funds [59].

*Policy advice 2*. According to the analysis of governmental subsidies, we conclude that governments need to continue increasing the awareness of the role of supervision. Governments should clearly understand the advantages of low carbon investment in manufacturing industries, and, at the same time, establish their own position in the incentive mechanism. To implement accurate supervision, it is necessary to improve the social supervision system. In addition, a complete database can be established to give a unified record of investors’ strategies so that their past behaviors can be given priority consideration when assessing investment decisions.

### 5.3. Sensitivity Analysis of Stable Points

**Figure 5:**
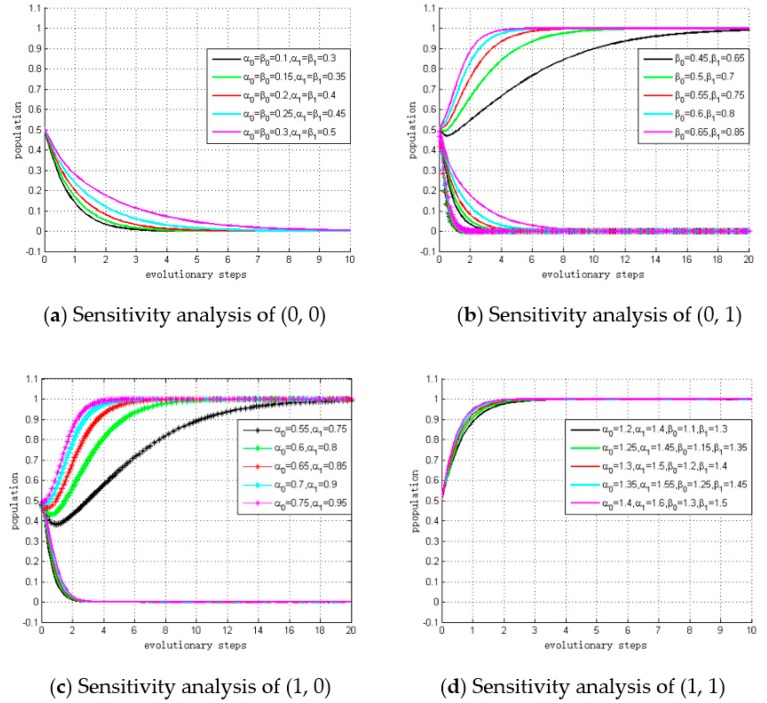
Simulation results of sensitivity analysis.

To examine whether the ESS results are robust to the change of profit growth coefficients or not, we conduct sensitivity analysis for different evolutionarily stable points. The initial population (x,y) is characterized as (0.51, 0.49), and we set the values of $\alpha_{0}$, $\alpha_{1}$, $\beta_{0}$ and $\beta_{1}$ vary within a fixed range, which are summarized in Table 8. The simulation results are depicted in Figure 5.

#### 5.3.1. Sensitivity Analysis of Stable Point (0, 0)

The simulation results are depicted in panel (a) of Figure 5. As shown, the lines spread over a much wider area than in the scenario in panel (d) of Figure 5, which means this scenario is more sensitive to the changes of the profit growth coefficients. In addition, it can be observed that with a smaller profit growth coefficient, fewer steps are required to reach ESS. In other words, it also implies the lower the profit from low carbon investment is, the larger the probability of making the strategic choice of “not invest” becomes.

#### 5.3.2. Sensitivity Analysis of Stable Point (0, 1)

Next, we conducted a sensitivity analysis of stable point (0, 1). Panel (b) in Figure 5 depicts the simulation results. As shown, it takes fewer steps for larger $\beta_{0}$ and $\beta_{1}$ values to reach the ESS, which means the convergent speed is faster and implies that when the profit growth coefficients of manufacturers increase, the probability of making the strategic choice of “invest” becomes larger.

Comparing the sensitivity analysis results shown in panels (a) and (b) of Figure 5, we find that if manufacturers choose “invest”, it takes fewer steps for suppliers to reach the ESS. This implies that if only one side of the players choose “invest”, the convergent speed of evolution trend will be faster. Moreover, the other side will quickly adjust their strategic choice to “not invest” and want to free ride.

#### 5.3.3. Sensitivity Analysis of Stable Point (1, 0)

Panel (c) in Figure 5 shows the simulation results of stable point (1, 0). As shown, it takes fewer steps for larger $\alpha_{0}$ and $\alpha_{1}$ to reach the ESS, which means the convergent speed is faster and when the profit growth coefficients of the suppliers increase, the probability of making the strategic choice of “invest” becomes larger.

In this scenario, the convergent speed of the manufacturers’ strategic choices is not particularly sensitive to the changes in the profit growth coefficients. Similar to the sensitivity analysis of (0, 1), this implies that manufacturers will choose “not invest” and want to free ride on suppliers.

#### 5.3.4. Sensitivity Analysis of Stable Point (1, 1)

Panel (d) in Figure 5 depicts the simulation result. As shown, the lines spread over a much narrower area than the other three scenarios, which means that this scenario is less sensitive to the changes in profit growth coefficients.

From a business perspective, as the profit growth coefficients increase to a critical level, the profits from low carbon investment are higher than the investment costs and profits from riding. Therefore, suppliers and manufacturers will eventually choose “invest”. The change of profit growth coefficients has no significant impacts on the convergent speed and evolution trend.

Based on the sensitivity simulation results, we propose further policy advice:

*Policy advice 3*. According to the sensitivity analysis simulation, the subtle variance of profit growth coefficients has more significant impacts on the evolutionary trend and convergent speed when the profit growth coefficients are small. Therefore, the economic situation of our case suggests that the primary task for Suntech and BYD is to take various measures to increase the profits from low carbon investment, and thus, policy advice 1 is preferable. 

As the profit growth coefficients increase, one side of the game players might choose “invest”. However, the other side may want to free ride. In this scenario, the incentive mechanism based on governmental subsidies is more important. Governments should reward and support those participants who persist in implementing low carbon investments, and guide suppliers and manufacturers to transform their investment behavior and awareness. Moreover, the power of social organizations should be used to supplement government regulations. This could include relaxing approval conditions to give legality and authority to related entities and supporting different low carbon investment activities organized by the associations through financial subsidies and social donations.

As the profit growth coefficients increase to a critical level, the low carbon raw material and low carbon products can bring expected profits. Therefore, both suppliers and manufacturers will be willing to invest in low carbon development for profit maximization. This is the optimal scenario, and we do not need to take extra measures.

### 5.4. Simulation of Impacts on ESS

#### 5.4.1. Impacts of $\alpha_{0}$, $\alpha_{1}$, $\beta_{0}$, and $\beta_{1}$ on ESS

**Figure 6:**
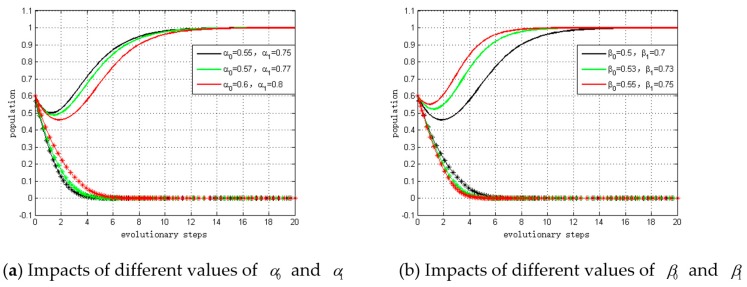
Impacts of profit growth coefficients on ESS.

We set the values of $\alpha_{0}$ to $\alpha_{0}$ = 0.55, 0.57, and 0.6. The values of $\alpha_{1}$ were defined as $\alpha_{1}$ = 0.75, 0.77, and 0.8. The initial values of $B_{S}$, $B_{M}$, $\xi_{S}$, $\xi_{M}$, $C_{S}$ and $C_{M}$ remained fixed, as defined in Section 5.1. The evolutionary trends under differing values of variables were compared and the simulation results are shown in panel (a) of Figure 6. The dotted lines represent suppliers, and the solid lines represent manufacturers. The ESS is (not invest, invest). From panel (a) in Figure 5, it can be observed that with smaller $\alpha_{0}$ and $\alpha_{1}$, fewer steps are required to reach ESS.

Similarly, we defined the values of $\beta_{0}$ as $\beta_{0}$ = 0.5, 0.53, and 0.55. The values of $\beta_{1}$ were defined as $\beta_{1}$ = 0.7, 0.73, and 0.75. The simulation results are shown in in panel (b) of Figure 6. From panel (b) in Figure 5, we can conclude that with larger $\beta_{0}$ and $\beta_{1}$ values, there is a higher probability of converging to (0, 1), which is consistent with and supports our theoretical analysis.

#### 5.4.2. Impacts of $C_{S}$ and $C_{M}$ on ESS

**Figure 7:**
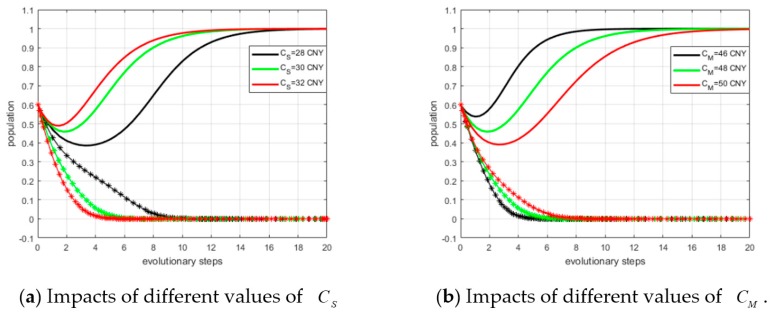
Impacts of investment costs on ESS.

By setting $\alpha_{0}$ = 0.6, $\alpha_{1}$ = 0.8, $\beta_{0}$ = 0.5, and $\beta_{1}$ = 0.7, we defined the values of $C_{S}$ as $C_{S}$ = 28 CNY, 30 CNY, and 32 CNY. The simulation results are depicted in panel (a) of Figure 7. It can be observed with a larger $C_{S}$ value, fewer steps are required to arrive at the ESS. Similarly, we defined $C_{M}$ as 46 CNY, 48 CNY, and 50 CNY, respectively. The simulation results are shown in panel (b) of Figure 7. From the simulation results, it can be concluded that the smaller $C_{M}$ is, the higher the probability of converging to (0, 1) is. The simulation results are also consistent with and support our theoretical analysis.

#### 5.4.3. Impacts of $B_{S}$ and $B_{M}$ on ESS

**Figure 8:**
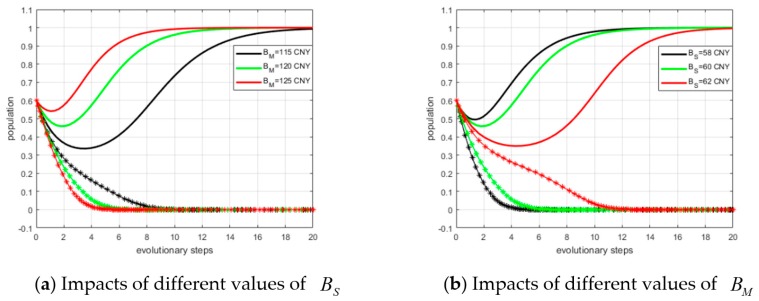
Impacts of original profits on ESS.

By setting $\alpha_{0}$ = 0.6, $\alpha_{1}$ = 0.8, $\beta_{0}$ = 0.5, and $\beta_{1}$ = 0.7, we defined the values of $B_{S}$ as $B_{S}$ = 58 CNY, 60 CNY, and 62 CNY. The simulation results are shown in panel (a) of Figure 8. It can be observed that with a smaller $B_{S}$ value, fewer steps are required to reach ESS. Next, we assumed $B_{M}$ = 115 CNY, 120 CNY, and 125 CNY. The simulation results are shown in panel (b) of Figure 8. Similarly, we can conclude with a larger $B_{M}$, fewer steps are required to reach ESS.

#### 5.4.4. Impacts of $\xi_{S}$ and $\xi_{M}$ on ESS

**Figure 9:**
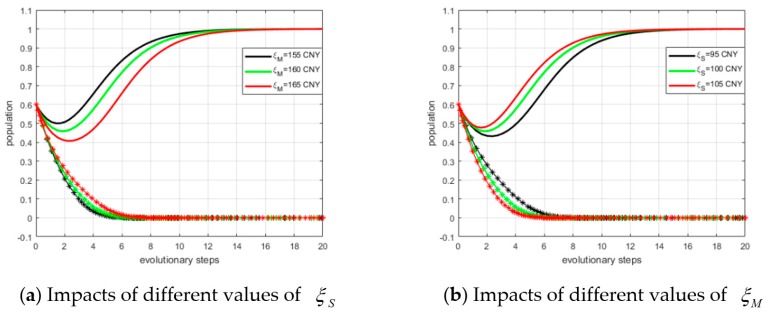
Impacts of profits from free riding on ESS.

We defined the value of $\xi_{S}$ as $\xi_{S}$ = 95 CNY, 100 CNY, and 105 CNY. The simulation results are shown in panel (a) of Figure 9. It can be observed that with a larger $\xi_{S}$ value, fewer steps are required to reach ESS. Next, we set $\xi_{M}$ to 155 CNY, 160 CNY, and 165 CNY. The simulation results are shown in panel (b) of Figure 9. Similarly, we concluded that with a smaller $\xi_{M}$, fewer steps are required to reach ESS. In other words, the probability of converging to the equilibrium point is higher.

Based on the simulations results of impacts on ESS, we also give advice for low carbon investment in supply chains:

*Policy advice 4.* Based on the above analysis and simulation results, the convergent speed appears to be more sensitive to the changes in investment costs $\left(C_{S},C_{M}\right)$ and original profits $\left(B_{S},B_{M}\right)$. In order to increase the convergent speed of the evolution trend to “invest”, the first step to motivate low carbon investment is to reduce the investment costs. This requires the government to promote and clarify the related corporate responsibilities to suppliers and manufacturers. Additionally, the cooperation among regulatory authorities should be strengthened. Furthermore, governments should take some measures to lower the price of high-carbon products and propose some rules to punish free riding behavior. These countermeasures could decrease the convergent speed to “not invest”.

According to the simulation of ESS and the sensitivity analysis, we conclude that the first step towards motivating low carbon investment is reducing the investment costs of suppliers and manufacturers. When the profit from low carbon strategies is small, attracting more consumers and expanding the market are more important for the profit growth coefficients. As the profit growth coefficients increase to a certain level, the game players want to free ride on the other side. In this scenario, the incentive (punishment) mechanism based on governmental subsidies has a more significant impact on the strategic choices of investors. When the profit from low carbon investments is large enough, both suppliers and manufacturers are willing to choose “invest”. This is the optimal ESS, and governments do not take any extra measures.

## 6. Conclusions

This paper uses a quantity-setting duopoly evolutionary game model to investigate when suppliers and manufacturers choose the optimal strategy for low carbon investment. We examine the conditions under which the chosen strategy is an ESS. Additionally, we verified the theoretical results with a numerical simulation. Different from previous research, our model analyzes the business problem of low carbon investment from the evolutionary perspective, and obtain several novel results via a theoretical analysis and numerical simulation:

(1) The strategic choice of low carbon investment by suppliers and manufacturers in supply chains is correlated with profit growth coefficients, investment costs, profits from free riding, and governmental subsidies, which is consistent with previous research.
(2) From the perspective of evolutionary analysis, the subtle variance of profit growth coefficients has more significant impacts on the evolutionary trend and convergence speed when the profit growth coefficients are small. As the profit growth coefficients increase, one side of game players will choose “invest”, and the other side want to free ride. In this scenario, the incentive mechanism based on governmental subsidies is more important. When the profit growth coefficients increase to a critical level, both suppliers and manufacturers are willing to choose “invest”. In this situation, the change of profit growth coefficients has slight impacts on the evolutionary trend and convergence speed. We only need to take some measures to ensure the sustainability of low carbon investment.
(3) When free riding is present, the probability of choosing (invest, not invest) or (not invest, invest) is correlated with the variables of original profits, profit growth coefficients, investment costs and profits from free riding. According to the simulation analysis, we find that the investment costs and original profits have more significant impacts on evolutionary trend and convergence speed.
(4) According to the model analysis, incentive based on governmental subsidies is effective to motivate low carbon investment and avoid free riding in supply chains. However, the investment decision might not be assessed precisely for the reason of limited budgets. In this scenario, free riding is still present.

In summary, our results show that profits growth coefficients, investment costs and profits from free riding all have impacts on the strategic choice of game players. However, these factors have different impact degrees in different stages of evolutionary process. For example, the profits growth coefficients have more significant impacts on evolutionary trend when they are small. Additionally, different factors have different impacts on the convergent speed of evolutionary process. For example, investment costs have more significant impacts than other factors when free riding is present. Based on the above analysis, an incentive mechanism based on governmental subsidies should be developed to motivate suppliers and manufacturers to choose “invest” and ensure that it is the only ESS.

Our study has several limitations that must be addressed. First, one could instead use an evolutionary game model for strategy choice based on a nonlinear demand function. It would be very interesting to compare those results with ours (but it would be very complicated to analyze). Second, a scenario involving an increased demand for low carbon products might be considered as this will affect the evolutionary path of the strategies. Finally, an interesting issue to address in future work is how other factors (e.g., the advertising investment of game players) affect the evolution of the strategic choice of low carbon investment.

## Figures and Tables

**Table 1:** Summary of notations.

Symbol	Description
$B_{S}$	Profits of suppliers if both players do not invest, $B_{S}>0$
$B_{M}$	Profits of manufacturers if both players do not invest, $B_{M}>0$
$C_{S}$	Investment costs of suppliers, $C_{S}>0$
$C_{M}$	Investment costs of manufacturers, $C_{M}>0$
$\xi_{S}$	Profits of suppliers from free riding, $\xi_{S}>B_{S}>0$
$\xi_{M}$	Profits of manufacturers from free riding, $\xi_{M}>B_{M}>0$
$\alpha_{0}$	Profit growth coefficient of suppliers if only suppliers invest, $\alpha_{0}>0$
$\alpha_{1}$	Profit growth coefficient of suppliers if both players invest, $\alpha_{1}>\alpha_{0}>0$
$\beta_{0}$	Profit growth coefficient of manufacturers if only manufacturers invest, $\beta_{0}>0$
$\beta_{1}$	Profit growth coefficient of manufacturers if both players invest, $\beta_{1}>\beta_{0}>0$

**Table 2:** The payoff matrix.

Suppliers	Manufacturers	
	Invest	Not Invest
Invest	$(1+\alpha_{1})B_{S}-C_{S}$, $(1+\beta_{1})B_{M}-C_{M}$	$(1+\alpha_{0})B_{S}-C_{S}$, $\xi_{M}$
Not Invest	$\xi_{S}$, $(1+\beta_{0})B_{M}-C_{M}$	$B_{S}$, $B_{M}$

**Table 3:** Values of equilibrium points.

Equilibrium Points	$a_{11}$	$a_{12}$	$a_{21}$	$a_{22}$
(0, 0)	$\alpha_{0}B_{S}-C_{S}$	0	0	$\beta_{0}B_{M}-C_{M}$
(0, 1)	$\alpha_{1}B_{S}-C_{S}-\xi_{S}+B_{S}$	0	0	$-(\beta_{0}B_{M}-C_{M})$
(1, 0)	$-(\alpha_{0}B_{S}-C_{S})$	0	0	$\beta_{1}B_{M}-C_{M}-\xi_{M}+B_{M}$
(1, 1)	$-(\alpha_{1}B_{S}-C_{S}-\xi_{S}+B_{S})$	0	0	$-(\beta_{1}B_{M}-C_{M}-\xi_{M}+B_{M})$
(A, B)	0	$a_{12}(A,B)$	$a_{21}(A,B)$	0

**Table 4:** Impacts on ESS (Evolutionary Stable Strategy) when variables change.

Parameter Change	$S_{M}$ ($S_{N}$)	ESS
$\alpha_{0}$↓, $\alpha_{1}$↓	↑(↓)	(not invest, invest)
$\beta_{0}$↑, $\beta_{1}$↑	↑(↓)	(not invest, invest)
$B_{S}$↓	↑(↓)	(not invest, invest)
$B_{M}$↑	↑(↓)	(not invest, invest)
$C_{S}$↑	↑(↓)	(not invest, invest)
$C_{M}$↓	↑(↓)	(not invest, invest)
$\xi_{S}$↑	↑(↓)	(not invest, invest)
$\xi_{M}$↓	↑(↓)	(not invest, invest)

**Table 5:** The payoff matrix under incentive mechanism.

Suppliers	Manufacturers	
	Invest	Not Invest
invest	$(1+\alpha_{1})B_{S}-C_{S}$, $(1+\beta_{1})B_{M}-C_{M}$	$(1+\alpha_{0})B_{S}-C_{S}+K$, $\xi_{M}-K$
not invest	$\xi_{S}-K$, $(1+\beta_{0})B_{M}-C_{M}+K$	$B_{S}$, $B_{M}$

**Table 6:** Initial values of parameters.

Parameters	$B_{S}$	$B_{M}$	$C_{S}$	$C_{M}$	$\xi_{S}$	$\xi_{M}$
Values	60	120	30	48	100	160

**Table 7:** Different values of $\alpha_{0}$, $\alpha_{1}$, $\beta_{0}$, $\beta_{1}$ and ESSs.

$\alpha_{0}$	$\alpha_{1}$	$\beta_{0}$	$\beta_{1}$	ESS
0.25	0.45	0.25	0.45	(not invest, not invest)
0.25	0.45	0.5	0.7	(not invest, invest)
0.6	0.8	0.25	0.45	(invest, not invest)
1.2	1.4	1.1	1.3	(invest, invest)

**Table 8:** Different values of $\alpha_{0}$, $\alpha_{1}$, $\beta_{0}$ and $\beta_{1}$ for the sensitivity analysis.

**Evolutionarily Stable Point: (0, 0)**				**Evolutionarily Stable Point: (0, 1)**			
$\alpha_{0}$	$\alpha_{1}$	$\beta_{0}$	$\beta_{1}$	$\alpha_{0}$	$\alpha_{1}$	$\beta_{0}$	$\beta_{1}$
0.1	0.3	0.1	0.3	0.1	0.3	0.45	0.65
0.15	0.35	0.15	0.35	0.15	0.35	0.5	0.7
0.2	0.4	0.2	0.4	0.2	0.4	0.55	0.75
0.25	0.45	0.25	0.45	0.25	0.45	0.6	0.8
0.3	0.5	0.3	0.5	0.3	0.5	0.3	0.85
**Evolutionarily Stable Point: (1, 0)**				**Evolutionarily Stable Point: (1, 1)**			
$\alpha_{0}$	$\alpha_{1}$	$\beta_{0}$	$\beta_{1}$	$\alpha_{0}$	$\alpha_{1}$	$\beta_{0}$	$\beta_{1}$
0.55	0.75	0.1	0.3	1.2	1.4	1.1	1.3
0.6	0.8	0.15	0.35	1.25	1.45	1.15	1.35
0.65	0.85	0.2	0.4	1.3	1.5	1.2	1.4
0.7	0.9	0.25	0.45	1.35	1.55	1.25	1.45
0.75	0.95	0.3	0.5	1.4	1.6	1.3	1.5

## Appendix A

If only supplier choose to “invest”, we define the earnings from low carbon investment as $E_{SI}$. If both players choose to “invest”, we define the earnings from low carbon investment as $E_{SB}$. Therefore, if $0<\alpha_{0}<\frac{C_{S}}{B_{S}}$ and $\alpha_{0}<\alpha_{1}<\frac{\xi_{S}+C_{S}-B_{S}}{B_{S}}$, we find that:

(A1) $$E_{SI}=(1+\alpha_{0})B_{S}-C_{S}<(1+\frac{C_{S}}{B_{S}})B_{S}-C_{S}=B_{S},\;E_{SB}=(1+\alpha_{1})B_{S}-C_{S}<(1+\frac{\xi_{S}+C_{S}-B_{S}}{B_{S}})B_{S}-C_{S}=\xi_{S}$$

Similarly, if only manufacturers choose to “invest”, we define the earnings from low carbon investment as $E_{MI}$. If both players choose to “invest”, we define the earnings from low carbon investment as $E_{MB}$. Therefore, if $0<\beta_{0}<\frac{C_{M}}{B_{M}}$, $\beta_{0}<\beta_{1}<\frac{\xi_{M}+C_{M}-B_{M}}{B_{M}}$, we find that:

(A2) $$E_{MI}=(1+\beta_{0})B_{M}-C_{M}<(1+\frac{C_{M}}{B_{M}})B_{M}-C_{M}=B_{M},\;E_{MB}=(1+\beta_{1})B_{M}-C_{M}<(1+\frac{\xi_{M}+C_{M}-B_{M}}{B_{M}})B_{M}-C_{M}=\xi_{M}$$

In this scenario, the expected profit of suppliers and manufacturers is less than the profit of choosing “not invest” and that from free riding. Therefore, both players will choose (not invest, not invest).

## Appendix B

If $\frac{C_{M}}{B_{M}}<\beta_{0}<\beta_{1}<\frac{\xi_{M}+C_{M}-B_{M}}{B_{M}}$, we find that:

(A3) $$E_{MI}=(1+\beta_{0})B_{M}-C_{M}>(1+\frac{C_{M}}{B_{M}})B_{M}-C_{M}=B_{M},\;E_{MB}=(1+\beta_{1})B_{M}-C_{M}<(1+\frac{\xi_{M}+C_{M}-B_{M}}{B_{M}})B_{M}-C_{M}=\xi_{M}$$

As a result, the expected profit of manufacturers is higher than the profit of choosing “not invest”, but lower than the profit from free riding. The expected profit of suppliers is the same as under Proposition 1. Therefore, suppliers and manufacturers will choose (not invest, invest).

## Appendix C

If $\frac{C_{S}}{B_{S}}<\alpha_{0}<\alpha_{1}<\frac{\xi_{S}+C_{S}-B_{S}}{B_{S}}$, we find that:

(A4) $$E_{SI}=(1+\alpha_{0})B_{S}-C_{S}>(1+\frac{C_{S}}{B_{S}})B_{S}-C_{S}=B_{S},\;E_{SB}=(1+\alpha_{1})B_{S}-C_{S}<(1+\frac{\xi_{S}+C_{S}-B_{S}}{B_{S}})B_{S}-C_{S}=\xi_{S}$$

As a result, the expected profit of suppliers is higher than the profit of choosing “not invest”, but lower than the profit from free riding. The expected profit of manufacturers is same as under Proposition 1. Therefore, suppliers and manufacturers will choose (invest, not invest).

## Appendix D

If $\frac{C_{S}}{B_{S}}<\alpha_{0}<\alpha_{1}<\frac{\xi_{S}+C_{S}-B_{S}}{B_{S}}$,

(A5) $$E_{SI}=(1+\alpha_{0})B_{S}-C_{S}>(1+\frac{C_{S}}{B_{S}})B_{S}-C_{S}=B_{S},\;E_{SB}=(1+\alpha_{1})B_{S}-C_{S}<(1+\frac{\xi_{S}+C_{S}-B_{S}}{B_{S}})B_{S}-C_{S}=\xi_{S}$$

If $\frac{C_{M}}{B_{M}}<\beta_{0}<\beta_{1}<\frac{\xi_{M}+C_{M}-B_{M}}{B_{M}}$,

(A6) $$E_{MI}=(1+\beta_{0})B_{M}-C_{M}>(1+\frac{C_{M}}{B_{M}})B_{M}-C_{M}=B_{M},\;E_{MB}=(1+\beta_{1})B_{M}-C_{M}<(1+\frac{\xi_{M}+C_{M}-B_{M}}{B_{M}})B_{M}-C_{M}=\xi_{M}$$

As a result, if either of game players chooses “not invest”, the other one will choose “invest” for $E_{SI}>B_{S}$ and $E_{MI}>B_{M}$. However, they will not choose “invest” simultaneously because $E_{SB}<\xi_{S}$ and $E_{MB}<\xi_{M}$. The profit from free riding is higher than the profit when both players choose ‘invest’. Therefore, the ESS profile can be either (not invest, invest) or (invest, not invest).

## Appendix E

If $\frac{\xi_{S}+C_{S}-B_{S}}{B_{S}}<\alpha_{0}<\alpha_{1}$, we find that:

(A7) $$E_{SI}=(1+\alpha_{0})B_{S}-C_{S}>(1+\frac{\xi_{S}+C_{S}-B_{S}}{B_{S}})B_{S}-C_{S}=\xi_{S}$$

(A8) $$E_{SB}=(1+\alpha_{1})B_{S}-C_{S}>(1+\frac{\xi_{S}+C_{S}-B_{S}}{B_{S}})B_{S}-C_{S}=\xi_{S}$$

If $\frac{\xi_{M}+C_{M}-B_{M}}{B_{M}}<\beta_{0}<\beta_{1}$, we find that:

(A9) $$E_{MI}=(1+\beta_{0})B_{M}-C_{M}>(1+\frac{\xi_{M}+C_{M}-B_{M}}{B_{M}})B_{M}-C_{M}=\xi_{M}$$

(A10) $$E_{MB}=(1+\beta_{1})B_{M}-C_{M}>(1+\frac{\xi_{M}+C_{M}-B_{M}}{B_{M}})B_{M}-C_{M}=\xi_{M}$$

As a result, the expected profit of choosing “invest” is higher than the profit of choosing “not invest” and that from free riding. Therefore, both players will choose (invest, invest).

## Appendix F

The first-order conditions of $S_{M}$ to $\alpha_{0}$ and $\alpha_{1}$ can be written as follows:

(A11) $$\begin{array}{l} \frac{dS_{M}}{d\alpha_{0}}=-\frac{[\xi_{S}-(1+\alpha_{1})B_{S}+C_{S}]B_{S}}{2{\left[\xi_{S}-(\alpha_{1}-\alpha_{0}+1)B_{S}\right]}^{2}}<0\\ \frac{dS_{M}}{d\alpha_{1}}=-\frac{[\alpha_{0}B_{S}-C_{S}]B_{S}}{2{\left[\xi_{S}-(\alpha_{1}-\alpha_{0}+1)B_{S}\right]}^{2}}<0 \end{array}$$

We can find that the smaller $\alpha_{0}$ and $\alpha_{1}$ are, the larger $S_{M}$ is. The larger $\alpha_{0}$ and $\alpha_{1}$ are, the smaller $S_{M}$ is. Therefore, we can reach Conclusion 1.

## Appendix G

The first-order conditions of $S_{M}$ to $\beta_{0}$ and $\beta_{1}$ can be written as follows:

(A12) $$\begin{array}{l} \frac{dS_{M}}{d\beta_{0}}=\frac{[\xi_{M}-(1+\beta_{1})B_{M}+C_{M}]B_{M}}{2{\left[\xi_{M}-(\beta_{1}-\beta_{0}+1)B_{M}\right]}^{2}}>0\\ \frac{dS_{M}}{d\beta_{1}}=\frac{[\beta_{0}B_{M}-C_{M}]B_{M}}{2{\left[\xi_{M}-(\beta_{1}-\beta_{0}+1)B_{M}\right]}^{2}}>0 \end{array}$$

We can find that the smaller $\beta_{0}$ and $\beta_{1}$ are, the smaller $S_{M}$ is. The large $\beta_{0}$ and $\beta_{1}$ are, the larger $S_{M}$ is. Therefore, we can conclude Conclusion 2.

## Appendix H

The first-order condition of $S_{M}$ to $B_{S}$ can be written as follows:

(A13) $$\frac{dS_{M}}{dB_{S}}=\frac{-\alpha_{0}\xi_{S}+(\alpha_{1}-\alpha_{0}+1)C_{S}}{2{\left[\xi_{S}-(\alpha_{1}-\alpha_{0}+1)B_{S}\right]}^{2}}$$

According to $\frac{C_{S}}{B_{S}}<\alpha_{0}<\alpha_{1}<\frac{\xi_{S}+C_{S}-B_{S}}{B_{S}}$, $\frac{dS_{M}}{dB_{S}}$ can be written as follows:

(A14) $$\frac{dS_{M}}{dB_{S}}=\frac{-\alpha_{0}\xi_{S}+(\alpha_{1}-\alpha_{0}+1)C_{S}}{2{\left[\xi_{S}-(\alpha_{1}-\alpha_{0}+1)B_{S}\right]}^{2}}<\frac{-\alpha_{0}\xi_{S}+\frac{\xi_{S}}{B_{S}}C_{S}}{2{\left[\xi_{S}-(\alpha_{1}-\alpha_{0}+1)B_{S}\right]}^{2}}=\frac{-\xi_{S}(\alpha_{0}-\frac{C_{S}}{B_{S}})}{2{\left[\xi_{S}-(\alpha_{1}-\alpha_{0}+1)B_{S}\right]}^{2}}<0$$

Similarly, we know that $1+\beta_{1}-\beta_{0}<1+\beta_{1}-\frac{C_{M}}{B_{M}}<\frac{\xi_{M}}{B_{M}}$. Thus, $\frac{dS_{M}}{dB_{M}}$ can be written as follows:

(A15) $$\frac{dS_{M}}{dB_{H}}=\frac{\beta_{0}\xi_{M}-(\beta_{1}-\beta_{0}+1)C_{M}}{2{\left[\xi_{M}-(\alpha_{1}-\alpha_{0}+1)B_{M}\right]}^{2}}>\frac{\beta_{0}\xi_{M}-\frac{\xi_{M}}{B_{M}}C_{M}}{2{\left[\xi_{M}-(\alpha_{1}-\alpha_{0}+1)B_{M}\right]}^{2}}=\frac{\xi_{M}(\beta_{0}-\frac{C_{M}}{B_{M}})}{2{\left[\xi_{M}-(\alpha_{1}-\alpha_{0}+1)B_{M}\right]}^{2}}>0$$

We can find that the smaller $B_{S}$ is and larger $B_{M}$ is, the larger $S_{M}$ is. The larger $B_{S}$ is and smaller $B_{M}$ is, the smaller $S_{M}$ is. Therefore, we can conclude Conclusion 3.

## Appendix I

The first-order conditions of $S_{M}$ to $C_{S}$ and $C_{M}$ can be written as:

(A16) $$\begin{array}{l} \frac{dS_{M}}{dC_{S}}=\frac{1}{2[\xi_{S}-(\alpha_{1}-\alpha_{0}+1)B_{S}]}>0\\ \frac{dS_{M}}{dC_{M}}=-\frac{1}{2[\xi_{M}-(\beta_{1}-\beta_{0}+1)B_{M}]}<0 \end{array}$$

We can find that the smaller $C_{M}$ is and larger $C_{S}$ is, the larger $S_{M}$ is. The larger $C_{M}$ is and smaller $C_{S}$ is, the smaller $S_{M}$ is. Therefore, we can conclude Conclusion 4.

## Appendix J

The first-order conditions of $S_{M}$ to $\xi_{S}$ and $\xi_{M}$ can be written as follows:

(A17) $$\begin{array}{l} \frac{dS_{M}}{d\xi_{S}}=\frac{\alpha_{0}B_{S}-C_{S}}{2{\left[\xi_{S}-(\alpha_{1}-\alpha_{0}+1)B_{S}\right]}^{2}}>0\\ \frac{dS_{M}}{d\xi_{H}}=-\frac{\beta_{0}B_{H}-C_{H}}{2{\left[\xi_{H}-(\beta_{1}-\beta_{0}+1)B_{H}\right]}^{2}}<0 \end{array}$$

We can find that the smaller $\xi_{M}$ is and larger $\xi_{S}$ is, the larger $S_{M}$ is. The larger $\xi_{M}$ is and smaller $\xi_{S}$ is, the smaller $S_{M}$ is. Therefore, we can conclude Conclusion 5.
